# High-Speed Current *dq* PI Controller for Vector Controlled PMSM Drive

**DOI:** 10.1155/2014/709635

**Published:** 2014-01-16

**Authors:** Mohammad Marufuzzaman, Mamun Bin Ibne Reaz, Labonnah Farzana Rahman, Tae Gyu Chang

**Affiliations:** ^1^Department of Electrical, Electronics and Systems Engineering, Universiti Kebangsaan Malaysia, 43600 Bangi, Selangor, Malaysia; ^2^School of Electrical and Electronics Engineering, Chung-Ang University, Seoul 156-756, Republic of Korea

## Abstract

High-speed current controller for vector controlled permanent magnet synchronous motor (PMSM) is presented. The controller is developed based on modular design for faster calculation and uses fixed-point proportional-integral (PI) method for improved accuracy. Current *dq* controller is usually implemented in digital signal processor (DSP) based computer. However, DSP based solutions are reaching their physical limits, which are few microseconds. Besides, digital solutions suffer from high implementation cost. In this research, the overall controller is realizing in field programmable gate array (FPGA). FPGA implementation of the overall controlling algorithm will certainly trim down the execution time significantly to guarantee the steadiness of the motor. Agilent 16821A Logic Analyzer is employed to validate the result of the implemented design in FPGA. Experimental results indicate that the proposed current *dq* PI controller needs only 50 ns of execution time in 40 MHz clock, which is the lowest computational cycle for the era.

## 1. Introduction

In recent years, the progression of semiconductor power devices, magnetic materials, and control theories has made the permanent magnet synchronous motors (PMSM) most widely used in the high performance applications. PMSM drive performance mainly depends on the quick and precise response of the system, as well as on the robustness of the control strategy [1]. In order to achieve dynamic performance, the vector control, also known as field-oriented control (FOC), of the PMSM drive is employed [2]. In fact, FOC relies on the space vector pulse width modulation (SVPWM) control strategy. By using SVPWM, the PMSM control becomes almost the same as the DC motor control [3]. In the FOC PMSM drive, the *dq*-axis current control plays an important role in determining the overall system performance [4]. Therefore, an intelligent current controller claims meticulous consideration for the high performance FOC PMSM drive systems. Moreover, the current controllers should be designed first to ensure current regulation with adequate dynamics and zero steady-state error, irrespective of the reference signals behind them [5]. There exist several controllers for controlling currents in FOC PMSM drives [6–8]. In order to eliminate the steady-state error, the proportional-integral (PI) controller can also be used. Besides, a PI controller is very sensitive to step change of command speed, parameter variations, and load disturbances. Relatively simple implementation makes the PI controller most widely used for PMSM. Therefore, a real time self-automated hardware implementation of the PI controller, as well as FOC, is desired [8].

Most applications of FOC PMSM have a control structure consisting of an internal current feedback loop. The performance of the system mainly depends on the quality of the applied current control strategy. Therefore, current control is one of the most important subjects of modern power electronics [9]. In FOC PMSM drives, vector control scheme is used along with PWM inverters to provide effective control of the motor torque over wide speed ranges. Good performance of vector control is achieved only when a fast current control is realized. In high-power applications, despite the advances in power device technology, the switching frequency is limited due to switching losses. Low switching frequency causes an increase in current distortion, machine losses, and torque ripple [10]. Thus, the implementation of vector control requires current controllers with fast response and high accuracy in order to provide the optimal efficiency of the servo drive [11].

Better current control performance depends on how quick the computed switching states vector is applied without sampling period delay. Lin-Shi et al. presents the implementation of a hybrid control strategy applied to a PMSM drive [12]. The research implemented vector control, using two PI current *dq* controllers, in a DSpace DS1104 board with the Simulink environment. However, computing loops must be very short in order to reduce current ripple to an acceptable value. So, a large computing effort is required to achieve a suitable velocity. Sant and Rajagopal implement vector control of a PMSM with a hybrid fuzzy PI speed controller with switching functions [13]. These switching functions are very simple and effective and do not demand any extra computations to arrive at the hybrid fuzzy PI controller outputs. The research implemented a fuzzy-PI speed controller in the outer loop and two PI controllers for controlling currents in the inner loop. The implementation was done in a 100 W, 7A, PMSM with TMS320F2812 DSP. The reference speed in each case was set at 1500 rpm with a regulated DC bus voltage of 15 V. Another research by Jung et al. used a hybrid fuzzy PI controller for controlling current in a PMSM drive [14]. In this research, a fuzzy PI-type control scheme for a PMSM was presented to achieve a robust current control performance. The proposed current control strategy consists of a decoupling controller and a fuzzy PI controller in order to account for the nonlinearity of a PMSM model and to stabilize the decoupled dynamics. The scheme prototype is simulated for a 1HP PMSM servo system. El-Sousy implemented a PI controller for controlling currents in FOC PMSM drive [1]. The design consisted of a sliding-mode controller (SMC) in the feedback loop. In addition, an online-trained wavelet-neural-network controller was connected in parallel with the SMC to construct a robust wavelet-neural-network sliding-mode controller (RWNNSMC). The research proposed the RWNNSMC for PMSM drive systems under FOC, guaranteeing robustness in the presence of parameter uncertainties. The research demonstrated the application of SMC-2DOF I-PDC and WNNC control systems to control the rotor speed of the field-oriented PMSM drive system. All the aforementioned researches, however, were implemented in either a DSP or a microcontroller. However, these digital solutions are still limited for complex control algorithms. Though multiprocessors schemes or high performance DSPs can deal with such applications, the cost exceeds the benefits [15]. Moreover, the microprocessor-based solutions are presently reaching its physical limits, which is not less than few microseconds [16]. On the other hand, specific hardware technology such as field-programmable gate array (FPGA) has the advantages of wide parallelism, deep pipelining, and flexible memory architecture over DSP. Thus, FPGA based current *dq* PI controller can be considered as an appropriate solution for reducing the execution time.

At present simple computational circuits that require very low processing times are implemented in FPGA. Therefore, a high-speed computation is always a key concern for FPGA implementation, which means reduction of the execution time as well as clock cycles. In order to reduce the execution time, it is necessary to perform the tasks in a plain and simple way rather than using complex circuitry. The FPGA implementation of a current *dq* controller in FOC PMSM drive presents researchers with another challenge in two respects. One is reducing the execution time; the other is correctness of the output. Better accuracy with minimal execution time is a major concern in realizing current *dq* controllers in FPGA. Several researchers implement current *dq* controllers in FPGA [17–20]. Marufuzzaman et al. proposed the idea of implementing a high-speed current *dq* PI controller into FPGA. The research proposed the idea of realizing a high-speed current *dq* PI controller into FPGA but did not show any results [17]. Beguenance et al. showed the hardware implementation of a current controller in a FOC PMSM drive [18]. The research implemented the PI controller along with a decoupling method for controlling *dq*-axis current of a FOC PMSM drive. The scheme used an extended Kalman filter-based speed and flux observer and simple PI controller for current control. The overall controller computation time was in the order of microseconds at both 100 MHz and 8 MHz clock speed. However, the operations of the PI controller need more than 1 *μ*s time which means that the design is not implemented in nanoseconds range. Another research project from Naouar et al. implemented a FPGA based predictive current controller for a synchronous machine (SM) speed drive [19]. A limited switching frequency predictive current controller was considered in this research. Although the predictive current controller is complex, the research ensured the quasi-instantaneous computation of the switching states. However, its implementation required 106 latency times. This means that the overall computation time was 2.12 *μ*s for 50 MHz clock that is still higher compared to nanoseconds range solution. Research by Ying-Shieh and Ming-Hung defined two different submodules needed to implement the current controller of an FOC PMSM drive [20]. This research used an adaptive fuzzy controller for speed control and a PI controller for current control. The research showed that the *dq*-axis PI controller submodule could be accomplished in six steps. The design used a finite-state machine method to lower the usage of FPGA resources. The implementation was done in Nios II Embedded Processor IP with 864 logic elements for the current controller and coordinate transformation (CCCT). The operation of each step needed 40 ns in 25 MHz of clock; that means that completing the operation of CCCT required at least 0.24 *μ*s execution time. Even if this is less than 1 *μ*s further reduction of execution time along with good accuracy will certainly improve the current *dq* PI controller performances.

This research implemented PI controller for controlling *dq*-axis current of FOC PMSM drive. Instead of implementing in DSP based solution this research shows the FPGA implementation of current *dq* PI controller in Quartus II Altera environment. The FPGA implementation of this current *dq* PI controller is executed in very short time with a good accuracy. The method is tested in different clock frequency to ensure that the required clock cycle is similar. Besides, the overall design is validated in real time. The result is finally compared with the numerical calculation to show the accuracy of the output. This FPGA realization of current *dq* PI controller is a key element for a SoC FOC PMSM drive.

## 2. Current *dq* PI Controller Model for FOC PMSM Drive

FOC is a control procedure to operate the motor that results in fast dynamic response and energy efficient operation at all speeds. It commutates the motor by calculating voltage and current vectors based on motor current feedback. It maintains high efficiency over a wide operating range and allows for precise dynamic control of speed and torque. In FOC, motor currents and voltages are manipulated in the *dq* reference frame of the rotor. This means that the measured motor currents must be mathematically transformed from the three-phase static reference frame of the stator windings to the two-axis rotating *dq* reference frame, prior to the processing by the PI controllers.

**Figure 1:**
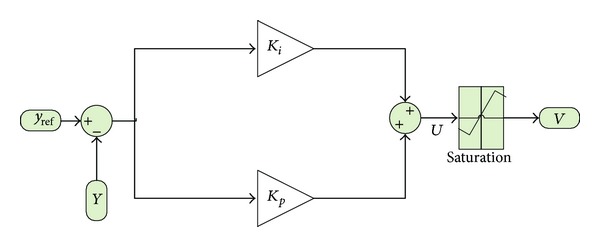
PI controller block diagram.

Figure 1 shows the basic block diagram of a PI controller. The error is directly sent to the current PI regulator. If the error has a very large value, the integrator will probably establish an excessive output. In this case the output result unwanted overshoot because of PI controller integral property.

Thus, the output of the PI controller should be limited to a certain value to prevent overshoots. To avert overflow the controller includes a saturator.

The mathematical model of the PI controller can be designed from Figure 1. Before passing through saturation, the output of this PI controller *u*(*t*_*n*_) can be written as

(1) $$\begin{array}{c} u\left(t_{n}\right)=K_{p}e\left(t_{n}\right)+I\left(t_{n}\right), \end{array}$$

where *K*_*p*_ is the proportional gain and *e*(*t*_*n*_) is the error which can be expressed as

(2) $$\begin{array}{c} e\left(t_{n}\right)=y_{\text{ref}}\left(t_{n}\right)-y\left(t_{n}\right), \end{array}$$

where *y*_ref_(*t*_*n*_) is the reference signal and *y*(*t*_*n*_) is the feedback signal.

Again the *n*th iteration *I*(*t*_*n*_) can be written as

(3) $$\begin{array}{c} I\left(t_{n}\right)=I\left(t_{n-1}\right)+K_{i}*\sum_{1}^{n}e\left(t_{n}\right), \end{array}$$

where *K*_*i*_ is the integral gain. All these gains are constants and depend on the system.

**Figure 2:**
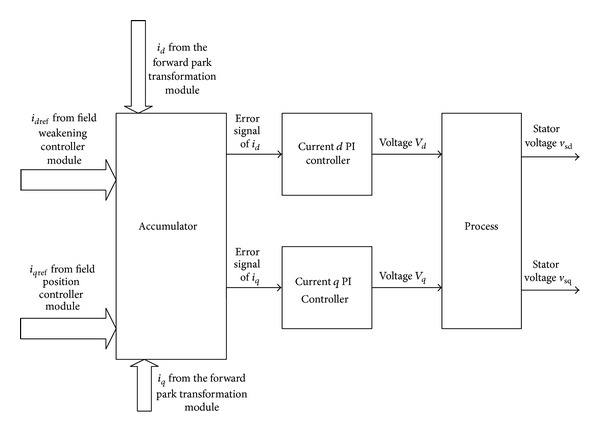
Current *dq* PI controller block diagram.

The overall block diagram of current *dq* PI controller module is shown in Figure 2. Initially input signal *i*_*q*ref_ is taken from the position controller and input signal *i*_*d*ref_ is taken from the field-weakening controller. The feedback *dq* current (*i*_*d*_, *i*_*q*_) signal is obtained from the forward park transformation. All these signals go through an accumulator, which will subtract the direct/quadrature current with the previously generated values to calculate the error signal. The outcome is passed to the PI controller. Finally, stator voltage (*V*_*sd*_,  *V*_sq_) signals are calculated from the error signal applying the following:

(4) $$\begin{array}{c} V_{\text{out}}=\left(K_{p}*\text{Error}\right)+K_{i\;}*\int_{1}^{n}\left(\text{Error}*dt\right), \end{array}$$

where *K*_*p*_ and *K*_*i*_ are proportional and integral gains that depend on the system.

There is also a saturation limit (*V*_max_,  *V*_min_) existing in this PI controller to control the *V*_out_. This protects the system from overshoots and undershoots. The phenomenon is called integrator antiwindup [21].

## 3. FPGA Implementation of the Proposed Current *dq* PI Controller

**Figure 3:**
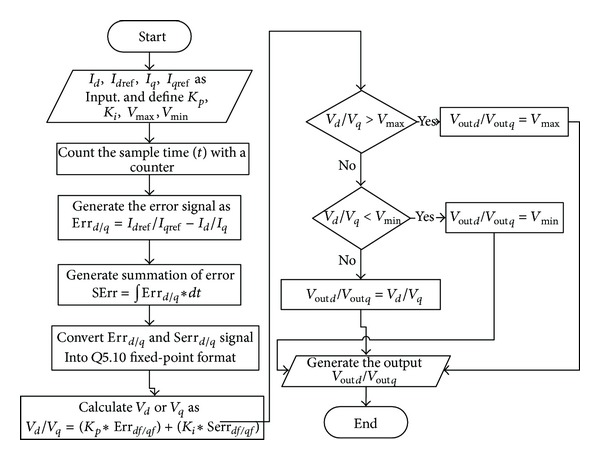
Process flow of current *dq* PI controller.

A high-end Altera Stratix IV EP4SGX230KF40C2 FPGA family based on Taiwan semiconductor manufacturing company (TSMC) 40 nm process has been used as target component for the implementation of the proposed controller. The chosen FPGA device surpasses all other high-end FPGAs, with the highest logic density, most transceivers, and lowest power requirements. It contains 182,400 logical elements and 14,625,792 memory bits. Some special features such as high-speed transceivers rated up to 8.5 Gbps, 600 MHz DSP performance, 600 MHz TriMatrix memory block, 1.6 Gbps LVDS channels, phase-locked loops (PLLs) for system clock management, and 1,067 Mbps (533 MHz) DDR3 memory interfaces support made Stratix IV a high-end solution for complex applications. According to the block diagram of Figure 2, the architecture of the overall system can be divided into two mirror components. Each component is partitioned into elementary modules. From a functional point of view, this partitioning makes the development process simpler.  Figure 3 shows the overall process flow of current *dq* PI controller implementation in FPGA. The processing starts by generating the *error*. Error is actually achieved by differentiating the two input current values. Then this error is converted to fixed-point format for further calculations. This is because the proportional and integral constants of the system are less than “1” and it can be represented in either floating point or fixed-point format [22]. Fixed-point format of decimal numbers is used to avoid the complex floating point calculations, which not only reduces the process time but also occupies less FPGA pins as well as logic elements [23]. Hence, FPGA implementation of fixed-point is more cost-effective compared to floating point arithmetic-based implementations [24]. Fixed-point representations require the programmer to create a virtual decimal place between two-bit locations for a given length of data [25]. A fixed-point binary number can be represented by *Qm*.*n* where *Q* is Texas Instruments representation for signed fixed-point numbers, *m* is the number of bits used, exclusive of the signed bit, and *n* is the number of fractional bits. The accuracy of floating point decimal values depends on the number of fractional bits. For more than 99.9% accuracy, fixed-point format *Q5.10* is sufficient. Thus fixed-point format *Q5.10* is used for FPGA implementation of current *dq* PI controller. The fixed-point format presents all the numbers in [−31.999, 31.999] range.

According to (4), the current *dq* PI controller has an integral part that is a known mathematical model. However, it is difficult or impossible to find an antiderivative, which is an elementary function. The complexity of integral made it intricate to implement in FPGA. Practical problem solving based on analyzing empirical, experimental, or measured data is required for solving this type of mathematical models. Numerical analysis and optimization methods are used to solve practical problems in computer science, business, engineering, and science. By applying numerical methods, integral calculation can be done in simple and faster way. Several formulae exist for solving the mathematical integration. The most simple and quickest method is the trapezium method. Thus, for quick calculation as well as accurate result, (4) of PI controller can be expressed in the following:

(5) Serrorn=Serror(n−1) +(12(errorn−error(n−1))×clockperiod),

where *S*error_*n*_ is the integral result and clock_period is the number of clock cycles needed for one iteration.

After calculating the error and *S*error, both of these values are converted to *Q5.10* fixed-point format to multiply with *K*_*p*_ and *K*_*i*_. Thus, (4) can be written as

(6) $$\begin{array}{c} V_{\text{out}(n)}=\left(K_{p}*{\text{error}\_f}_{n}\right)+\left(K_{i\;}*S\text{error}\_f_{n}\right), \end{array}$$

where error*_ f* and *s*error*_ f* are the fixed-point formatted value of error and *s*error, respectively.

Finally, the initial output voltage is checked with the maximum and minimum threshold voltages to control the saturation. Negative number multiplication is separately handled in this process for the negative floating-point result accuracy. The overall process is controlled by a global *reset* and an internal *ready* signal, which is synchronized with the clock.

The input/output signals of the FPGA implementation of current *dq* PI controller are shown in Table 1. The input current signals are 16 bits integer numbers, while the output is 16 bits *Q5.10* fixed-point format. Hence, a total of 66 pins are required for input and 32 pins are required for output signals.

In order to implement the task, several registers are needed for the FPGA implementation as shown in Table 2. Some system dependant parameters are also used for flexibility of the design. These parameters are defined as constants at the beginning of the design for ease of implementation.

## 4. Results and Discussion

**Figure 4:**
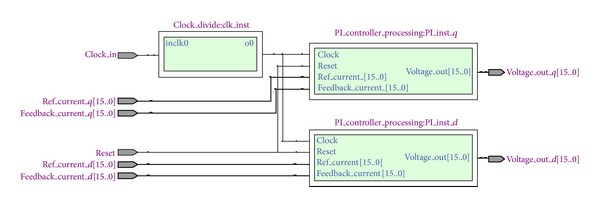
RTL view of current *dq* PI controller.

FPGA implementation of the current *dq* PI controller has been developed in Quartus II Altera environment. The hardware description language (HDL) used in this work is Verilog HDL as it is easy to understand and very similar to the most popular programming language, that is, C. The proposed current *dq* PI controller module has two mirror functions as well as a Phase Lock Loop (PLL). These two processing functions perform the same tasks with different data sets as shown in Figure 3. Each of these processing modules consists of three submodules, named error generator, PI regulator, and the limiter. In order to synchronize these two processing functions, *clock* and *reset* signal is used which is also depicted in Figure 4. The *clock* signal is actually the output of PLL for reducing overall execution time.

In Quartus II altera environment, it is possible to simulate the design even with the gate level delays. ModelSim Altera SE 10.0c is a widely used simulator for viewing and analyzing the simulation results of FPGA. Thus, it is used in this research to show the simulation output of this system. Figure 4 shows the Gate Level Simulation (GLS) of current *dq* PI controller of FOC PMSMS drives. The design runs in 40 MHz clock frequency as shown in Figure 4. So each clock period is required only 25 ns. In Figure 4 it is shown that if the reset signal is high, that is, “1,” the controller will not show any output. It is also clearly shown that the calculated output needs only 2 clock cycles for its execution which is only 50 ns.

The overall FPGA realization process of current *dq* PI controller implements the simplest design with minimal calculation. In addition, the overall process is partitioned into elementary modules and each module is working in parallel to reduce the execution time. The design uses registers level sensitivity instead of clock level sensitivity. This means that any processing blocks execution depend on changing in register values. Therefore, the design required less clock cycles. Moreover, as mentioned earlier the design is used fixed-point, which required less bits to represent values. That means that the register is also small and so the processing blocks work faster. Thus the overall execution time is reduced dramatically, which give a great benefit of implementing the SoC of FOC PMSM drive.

The design summery of the proposed current *dq* PI controller is shown in Table 3. According to the table, it is shown that the hardware implementation of current *dq* PI controller supports up to 40 MHz clock speed. It is also shown that the controller needs less than 1% of total logic elements as well as total combinational functions. Total registers used by this design are 254 which is less than 1% of the total registers of this device which means a small chunk of FPGA resources is used by the module. Most importantly, the hardware implementation does not occupy any memory blocks of FPGA. Therefore, FPGA cost of this proposed controller is very low. In other words, the proposed FPGA based controller is also suitable for cost sensitive applications.

As mentioned earlier, the test parameters are defined and are constant throughout the design process. It has been noted that variation in defining the parameters affects the accuracy of the proposed current *dq* PI controller. The variation of accuracy occurs because not all the decimal values can be represented in limited binary digits. Thus to validate the result, two different sets of parameters are defined for testing and compared with the mathematically calculated results. The first set of parameters defined for testing the design that is already mentioned earlier is as follows:

(7) $$\begin{array}{c} K_{p}=0.625,\;\;\;K_{i}=0.5,\\ V_{\max}=20,\;\;V_{\min}=2. \end{array}$$

The parameters are converted to *Q5.10* fixed-point format and defined at the beginning of the program. Five different sets of 16 bits random data are tested for verification. The hand calculation is done according to (6). For example, if *i*_*d*_ = 5 and *i*_*d*ref_ = 10 then the hand-calculated result would be as follows:

(8) error=idref−id=10−5  =5,serror=0+  12(5)∗4=10.Numerical  Result  (NR)=error∗Kp+serror∗Ki=5∗0.625+10∗0.5=8.125.

This hand calculation is actually done in an FLP model of MATLAB. In simulation results, which are shown in Figure 4, all outputs of the proposed module are multiplied by 1024 or 2^10^ according to fixed-point format. All of these values are represented in signed decimal numbers. Therefore, the output values need to be divided by 2^10^ to achieve the original results. The output values are limited within a specific range to prevent numerical overflow and alleviate windup phenomenon. Thus for the same set of test data, the simulated results would be as follows:

(9) $$\begin{array}{c} \text{Simulated}\;\text{Result}\;\left(\text{SR}\right)=\frac{8320}{2^{10}}=8.125. \end{array}$$

Comparing (8) with (9), it is obvious that the results are similar, which means that accuracy is 100%.

The defined parameters are now changed as follows:

(10) $$\begin{array}{c} K_{p}=0.6,\;\;\;K_{i}=0.5,\\ V_{\max}=20,\;\;V_{\min}=2. \end{array}$$

The GLS output using these set of parameters is shown in Figure 6.

Similarly, the hand calculation is done according to (6). For example if *i*_*d*_ = 5 and *i*_*d*ref_ = 10, then the hand calculated result would be as follows:

(11) error=idref−id=10−5=5,serror=0+12(5)∗4=10.Numerical  result  (NR)=error∗Kp+serror∗Ki=5∗0.6+10∗0.5=8.00.

**Figure 5:**
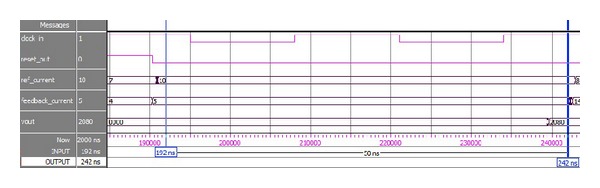
GLS output in 40 MHz clock frequency for *K*_*p*_ = 0.625.

Now considering Figure 5, the simulated results would be as follows:

(12) $$\begin{array}{c} \text{Simulated}\;\text{Result}\;\left(\text{SR}\right)=\frac{8190}{2^{10}}=7.99804687. \end{array}$$

Comparing (11) with (12), the accuracy of the module is measured:

(13) $$\begin{array}{c} \text{Accuracy}=\left(\frac{\text{SR}}{\text{NR}}\right)*100\%=99.98\%. \end{array}$$

**Figure 6:**
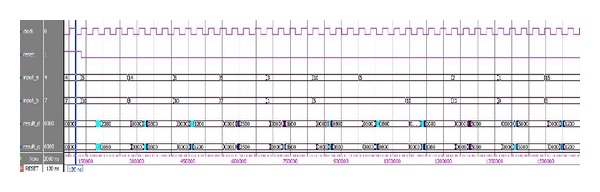
GLS output in 40 MHz clock frequency for *K*_*p*_ = 0.6.

**Figure 7:**
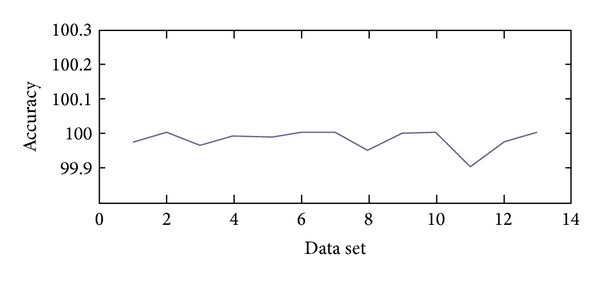
Measured accuracy of the proposed controller.

All those data sets are tested for verification and the accuracy chart is shown in Figure 6. The mean value of the accuracy is 99.98%. Therefore, form Figure 7 it is obvious that if the defined parameters can be represented within limited binary digits then the accuracy will be 100%; otherwise it will fall to 99.98%.

According to Figure 7, it is understandable that the results are similar though the module has some floating-point calculations. As the module used *Q5.10 *fixed-point formats so that the result is almost the same as decimal values. Again, the modular approach with each elementary module calculation made the results accurate. Instead of processing overall calculation, each of the iterations is performed at once. Negative number manipulation is another challenging task as it may produce wrong outcomes. In this design, negative fixed-point numbers are handled separately so that the correctness is ensured without influencing the execution time.

**Figure 8:**
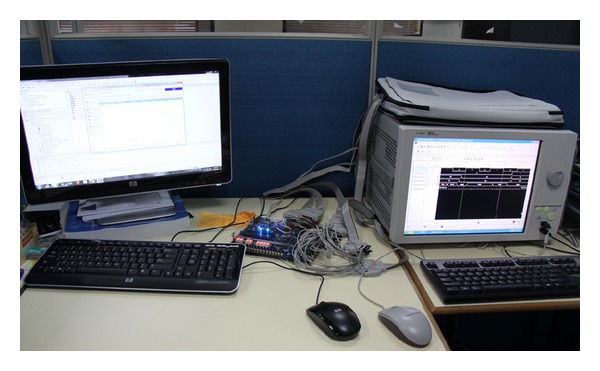
Photograph of experimental setup.

After implementing the design in FPGA, hardware testing was done. A photograph of experimental setup is shown in Figure 8. The experimental setup contains a logic analyzer, the DE4 FPGA board, and a computer. This research uses Agilent 16821A Logic Analyzer as it provide debugging, validating, and optimizing facility to digital systems in easier and faster way. Moreover, the device has built-in PG for generating test patterns as well as clock frequency.

**Figure 9:**
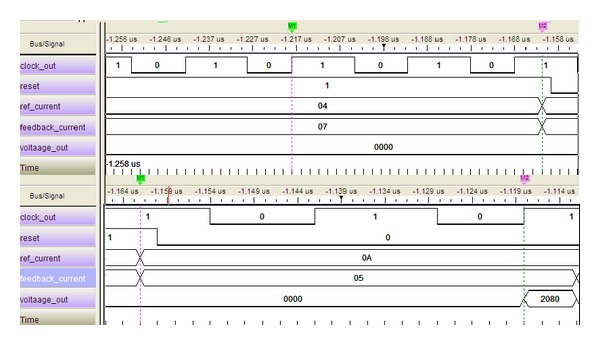
LA output in 40 MHz clock frequency for *K*_*p*_ = 0.625.

**Figure 10:**
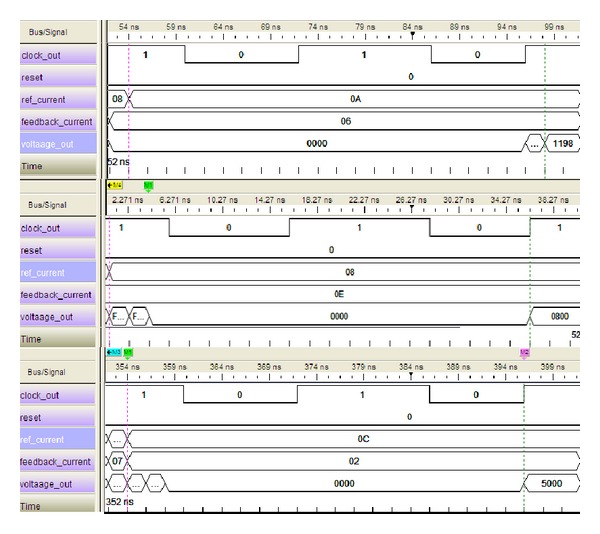
LA output in 40 MHz clock frequency for *K*_*p*_ = 0.6.

The simulation results of the hardware testing are shown in Figures 9 and 10. The test vectors are the same test data that has been defined for functional and gate-level simulations. From Figure 8, it is clear that the overall execution time required is less than 50 ns. In the reset condition, the controller does not produce any output. The calculated output is the same as the GLS output shown in Figure 5.

Similarly, the second sets of data are tested and shown in Figure 9. After processing in FPGA, the LA output shows the same results as the GLS. Moreover, the maximum and minimum condition also tested and the result is shown in Figure 9. The hardware testing clearly shows the reduced execution time of the proposed current *dq* PI controller. The clock frequency supported by this design is clearly sufficient for FOC PMSM drive. Thus, the design meets the design requirements of the overall controller.

FPGA realization of current *dq* PI controller for FOC PMSM drive is successfully completed and validated with other researches for comparison as shown in Table 4. Instead of using DSP based solution in [1, 12–14], this research proposed the FPGA realization of current *dq* PI controller. Moreover, it shows that this work can accomplish the transformation within 2 clock cycles which means that the execution time is as low as 50 ns in 40 MHz frequency. The execution time required in this proposed solution is much smaller than research from [18–20]. The proposed design required no memory resources as required in [20]. Although the proposed controller needs some floating point calculations; it provides good accuracy. 40 MHz clock frequency is more than enough for FOC PMSM drive system. Thus, from the comparison study it is observed that this proposed FPGA implementation of current *dq* PI controller is a faster and accurate solution for a real time current *dq* PI controller of FOC PMSM drive.

## 5. Conclusion

In this research, a current *dq* PI controller employing fixed-point is implemented in hardware. The hardware is realized based on a modular design along with a PLL, which simplifies the system as well as reducing the clock cycles. The experimental results indicate that the proposed hardware implementation requires only 50 ns or two clock cycles in the operating frequency of 40 MHz, which alleviates the performance of the current *dq* PI controller in terms of execution time. Comparison of the results with the findings from other research works shows that the method can offer a substantial reduction in execution time. Moreover, by using a fixed-point format, the proposed solution has produced more 99.98% accurate results, while the proposed controller occupies only 1182 logic elements and 254 registers. Thus, it consumes less than 1% logic elements and registers of the target FPGA. After implementing the proposed controller in FPGA, hardware testing is done, and the results are similar to the simulation results. The proposed hardware implementation of the current *dq* PI controller results in the improvement of the FOC PMSM drive system, which is considered a strong contender for building a SoC FOC PMSM drive.

## Figures and Tables

**Table 1:** IO signals of current *dq* PI controller.

Signal name	Input/output (bits)
Clock	INPUT
Reset	INPUT
ref_current_*d*	INPUT (16 bits)
feedback_current_*d*	INPUT (16 bits)
voltage_out_*d*	OUTPUT (16 bits)
ref_current_*q*	INPUT (16 bits)
feedback_current_*q*	INPUT (16 bits)
voltage_out_*q*	OUTPUT (16 bits)

**Table 2:** Major registers used in current *dq* PI controller.

Register name	Bits	Description
*K*_*p*_ (PARAMETER)	16	Proportional gain
*K*_*i*_ (PARAMETER)	16	Integral gain
*V*_max_ (PARAMETER)	16	Maximum limit of output voltage
*V*_min_ (PARAMETER)	16	Minimum limit of output voltage
ready	01	Enable processing
error	16	Store the error
error_*f*	16	Fixed-point format of error
sum_error	16	Integral of error
sum_error_*f*	16	Fixed-point format of sum_error
voltage_out_initial	16	Initial output voltage

**Table 3:** Design summery of current *dq* PI controller.

FPGA family	Stratix IV
Device	EP4SGX230KF40C2
Total logic elements	1182/182,400 (<1%)
Total combinational functions	1185/182,400 (<1%)
6 input functions 5 input functions 4 input functions ≤3 input functions	59 155 328 643
Total registers	254/182,400 (<1%)
Total block memory bits	0/14,625,792 (0%)
Clock	40 MHz
Total pins	98

**Table 4:** Performance comparison with other works.

	Realization	Execution time	Clock cycle	Other information
El-Sousy [1]	DSP	NA	NA	SMC for current control
Lin-Shi et al. [12]	DSP	NA	NA	Hybrid control
Sant [13]	DSP	NA	NA	PI controller
Jung et al. [14]	DSP	NA	NA	Hybrid fuzzy PI controller
Beguenane et al. [18]	FPGA	>1 *μ*s	—	PI controller with decoupling method
Naouar et al. [19]	FPGA	2.12 *μ*s	106	Predictive current controller
Kung and Tsai [20]	FPGA	240 ns	6	PI controller
This work	FPGA	50 ns	2	PI controller with fixed point
